# Ethyl 4-acetamido-3-acet­oxy-2-benzyl-3-methyl­butano­ate

**DOI:** 10.1107/S1600536811049440

**Published:** 2011-11-25

**Authors:** Guang Liang Wang, Ji Mei Zhang, Xin Zhang, Hao Xu, Gui Yun Duan

**Affiliations:** a Taishan Medical College, Tai an 271016, People’s Republic of China

## Abstract

The crystal structure of the title compound, C_18_H_25_NO_5_, is stabilized by inter­molecular N—H⋯O hydrogen bonds, which form inversion dimers. The ethyl group is disordered over two positions in a 0.651 (12):0.349 (12) ratio.

## Related literature

For the pharmacological activity of pyrrolidin-2-one compounds, see: Ichikawa & Kato (2001 ▶). For applications of related compounds, see: De Clercq (2004 ▶); Ge *et al.* (2009 ▶, 2011 ▶). The synthesis of the title compound was adapted from literature procedures for the preparation of closely related compounds, see: Bishop *et al.* (1991 ▶).
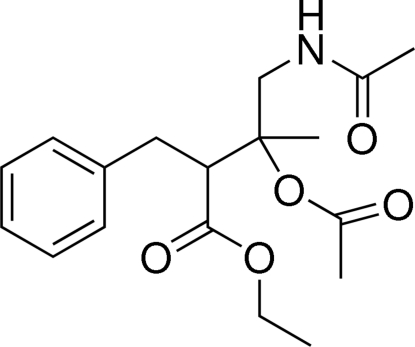


## Experimental

### 

#### Crystal data

C_18_H_25_NO_5_
*M* *_r_* = 335.39
Triclinic, 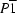

*a* = 9.7995 (18) Å
*b* = 10.0340 (19) Å
*c* = 10.481 (2) Å
α = 100.571 (3)°
β = 105.350 (3)°
γ = 107.957 (3)°
*V* = 905.1 (3) Å^3^
*Z* = 2
Mo *K*α radiation
μ = 0.09 mm^−1^
*T* = 293 K
0.24 × 0.19 × 0.16 mm

#### Data collection

Bruker SMART APEXII diffractometer
Absorption correction: multi-scan (*SADABS*; Bruker, 2005 ▶) *T*_min_ = 0.979, *T*_max_ = 0.986
4623 measured reflections
3167 independent reflections
2598 reflections with *I* > 2σ(*I*)
*R*_int_ = 0.016

#### Refinement

*R*[*F* ^2^ > 2σ(*F* ^2^)] = 0.045
*wR*(*F* ^2^) = 0.138
*S* = 1.06
3167 reflections
236 parameters
2 restraints
H-atom parameters constrained
Δρ_max_ = 0.20 e Å^−3^
Δρ_min_ = −0.19 e Å^−3^

### 

Data collection: *SMART* (Bruker, 2005 ▶); cell refinement: *SAINT* (Bruker, 2005 ▶); data reduction: *SAINT*; program(s) used to solve structure: *SHELXS97* (Sheldrick, 2008 ▶); program(s) used to refine structure: *SHELXL97* (Sheldrick, 2008 ▶); molecular graphics: *XP* in *SHELXTL* (Sheldrick, 2008 ▶); software used to prepare material for publication: *SHELXL97*.

## Supplementary Material

Crystal structure: contains datablock(s) I, global. DOI: 10.1107/S1600536811049440/fy2028sup1.cif

Structure factors: contains datablock(s) I. DOI: 10.1107/S1600536811049440/fy2028Isup2.hkl

Supplementary material file. DOI: 10.1107/S1600536811049440/fy2028Isup3.cml

Additional supplementary materials:  crystallographic information; 3D view; checkCIF report

## Figures and Tables

**Table 1:** Hydrogen-bond geometry (Å, °)

*D*—H⋯*A*	*D*—H	H⋯*A*	*D*⋯*A*	*D*—H⋯*A*
N1—H1⋯O4^i^	0.86	2.30	3.074 (2)	149

Symmetry code: (i) 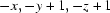.

**Fig. 1.**
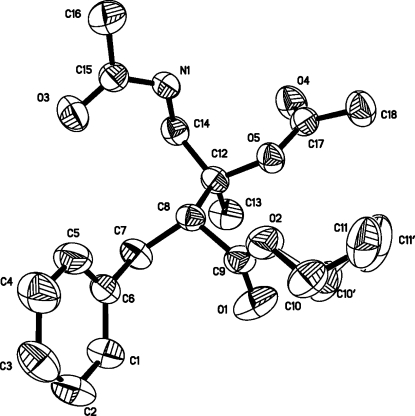
Molecular structure of the title compound, showing displacement ellipsoids drawn at the 50% probability level. Hydrogen atoms have been omitted for clarity.

**Fig. 2.**
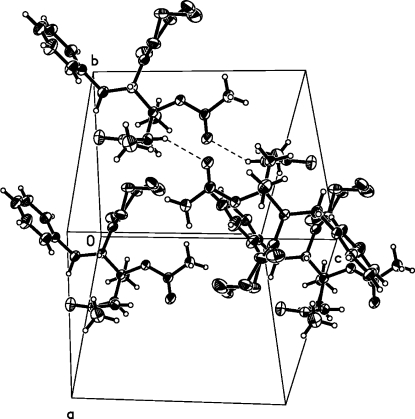
Crystal packing of the title compound. Thin dashed lines indicate the N1—H1···O4i hydrogen bond. Symmetry code: (i) –x, –y+1, –z+1.

**Fig. 3.**
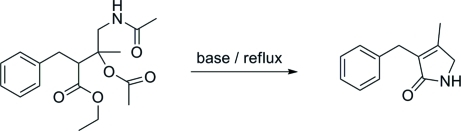
Reaction scheme showing the relationship of the title compound to pyrrolidin-2-ones.

## supplementary crystallographic information

### Comment

Synthesis of nitrogen-containing heterocyclic compounds has been a subject of great interest due to their widespread application in the agrochemical and pharmaceutical fields (Ge *et al.*; 2011, 2009). Some pyrrolidin-2-one derivatives which belong to this category have been of interest for their biological activities. Considerable effort has been devoted to the development of novel pyrrolidin-2-one compounds (De Clercq, 2004). We report herein the crystal structure of the title compound (Figs. 1 and 2) which is an important intermediate for the syntheses of pyrrolidin-2-ones (Fig. 3).

### Experimental

The synthesis of the title compound was adapted from literature procedures for the preparation of closely related compounds (Bishop *et al.*, 1991). A mixture of ethyl 3-oxobutanoate (0.1 mol), (chloromethyl)benzene (0.1 mol) and sodium ethanolate (0.15 mol) in ethanol (300 ml) was heated to reflux for 4 h. The product, ethyl 2-benzyl-3-oxobutanoate, was separated by column chromatography on silica gel (yield 76%). Ethyl 2-benzyl-3-oxobutanoate was reacted with HCN in ether below 15°C for 6 h. After removing the solvent, the residue was charged in a 500 ml autoclave. Then 50 g of Raney Ni and 300 ml of acetic anhydride were added to the autoclave. The mixture was reacted at 45°C under a hydrogen pressure of 2–3 MPa until the pressure reduction ceased. The Ni was removed by filtration and then the solvent was removed under reduced pressure. The final product was recrystallized from ethanol (yield 46%). Crystals of the product suitable for X-ray diffraction were obtained by slow evaporation of the solution of the product in ethanol at room temperature over 1 week.

### Refinement

All H atoms were placed in geometrically calculated positions and refined using a riding model with C—H = 0.97 Å (for CH_2_ groups) and 0.96 Å (for CH_3_ groups), and with N—H = 0.86 Å. Their isotropic displacement parameters were set to 1.2 times (1.5 times for CH_3_ groups) the equivalent displacement parameter of their parent atoms. Bond distances between the disordered C10—C11 and C10'—C11' atoms were restrained to 1.540 (3) Å.

### Crystal data

**Table:**

C_18_H_25_NO_5_	*Z* = 2
*M**_r_* = 335.39	*F*(000) = 360
Triclinic, *P*1	*D*_x_ = 1.231 Mg m^−^^3^
Hall symbol: -P 1	Mo *K*α radiation, λ = 0.71073 Å
*a* = 9.7995 (18) Å	Cell parameters from 2878 reflections
*b* = 10.0340 (19) Å	θ = 2.6–28.3°
*c* = 10.481 (2) Å	µ = 0.09 mm^−^^1^
α = 100.571 (3)°	*T* = 293 K
β = 105.350 (3)°	Block, colorless
γ = 107.957 (3)°	0.24 × 0.19 × 0.16 mm
*V* = 905.1 (3) Å^3^	

### Data collection

**Table:**

Bruker SMART APEXII diffractometer	3167 independent reflections
Radiation source: fine-focus sealed tube	2598 reflections with *I* > 2σ(*I*)
graphite	*R*_int_ = 0.016
φ and ω scans	θ_max_ = 25.1°, θ_min_ = 2.1°
Absorption correction: multi-scan (*SADABS*; Bruker, 2005)	*h* = −10→11
*T*_min_ = 0.979, *T*_max_ = 0.986	*k* = −11→10
4623 measured reflections	*l* = −10→12

### Refinement

**Table:**

Refinement on *F*^2^	Primary atom site location: structure-invariant direct methods
Least-squares matrix: full	Secondary atom site location: difference Fourier map
*R*[*F*^2^ > 2σ(*F*^2^)] = 0.045	Hydrogen site location: inferred from neighbouring sites
*wR*(*F*^2^) = 0.138	H-atom parameters constrained
*S* = 1.06	*w* = 1/[σ^2^(*F*_o_^2^) + (0.0739*P*)^2^ + 0.2116*P*] where *P* = (*F*_o_^2^ + 2*F*_c_^2^)/3
3167 reflections	(Δ/σ)_max_ < 0.001
236 parameters	Δρ_max_ = 0.20 e Å^−^^3^
2 restraints	Δρ_min_ = −0.19 e Å^−^^3^

### Special details

**Table:**

Geometry. All e.s.d.'s (except the e.s.d. in the dihedral angle between two l.s. planes) are estimated using the full covariance matrix. The cell e.s.d.'s are taken into account individually in the estimation of e.s.d.'s in distances, angles and torsion angles; correlations between e.s.d.'s in cell parameters are only used when they are defined by crystal symmetry. An approximate (isotropic) treatment of cell e.s.d.'s is used for estimating e.s.d.'s involving l.s. planes.
Refinement. Refinement of *F*^2^ against ALL reflections. The weighted *R*-factor *wR* and goodness of fit *S* are based on *F*^2^, conventional *R*-factors *R* are based on *F*, with *F* set to zero for negative *F*^2^. The threshold expression of *F*^2^ > σ(*F*^2^) is used only for calculating *R*-factors(gt) *etc*. and is not relevant to the choice of reflections for refinement. *R*-factors based on *F*^2^ are statistically about twice as large as those based on *F*, and *R*- factors based on ALL data will be even larger.

### Fractional atomic coordinates and isotropic or equivalent isotropic displacement parameters (Å^2^)

**Table:**

	*x*	*y*	*z*	*U*_iso_*/*U*_eq_	Occ. (<1)
O1	0.14359 (19)	0.04705 (16)	0.78101 (19)	0.0813 (5)	
O2	0.34401 (17)	0.22351 (16)	0.77960 (16)	0.0650 (4)	
O3	0.20424 (15)	0.64375 (16)	1.02379 (13)	0.0606 (4)	
O4	−0.09510 (15)	0.32515 (17)	0.46869 (13)	0.0614 (4)	
O5	0.09310 (13)	0.29479 (13)	0.62267 (11)	0.0451 (3)	
N1	0.12798 (17)	0.57192 (15)	0.79270 (15)	0.0456 (4)	
H1	0.1480	0.5898	0.7211	0.055*	
C1	0.2615 (2)	0.1673 (2)	1.1462 (2)	0.0556 (5)	
H1A	0.1808	0.0775	1.1024	0.067*	
C2	0.3832 (3)	0.1795 (3)	1.2567 (2)	0.0675 (6)	
H2	0.3837	0.0982	1.2872	0.081*	
C3	0.5025 (3)	0.3099 (3)	1.3211 (2)	0.0696 (6)	
H3	0.5837	0.3183	1.3963	0.083*	
C4	0.5024 (3)	0.4290 (3)	1.2746 (2)	0.0750 (7)	
H4	0.5849	0.5178	1.3168	0.090*	
C5	0.3802 (2)	0.4171 (2)	1.1653 (2)	0.0636 (6)	
H5	0.3804	0.4988	1.1353	0.076*	
C6	0.25803 (19)	0.28698 (19)	1.09999 (17)	0.0442 (4)	
C7	0.1239 (2)	0.2751 (2)	0.98167 (18)	0.0494 (4)	
H7A	0.0849	0.3488	1.0111	0.059*	
H7B	0.0434	0.1799	0.9579	0.059*	
C8	0.16401 (18)	0.29482 (18)	0.85328 (16)	0.0397 (4)	
H8	0.2507	0.3879	0.8806	0.048*	
C9	0.2136 (2)	0.1739 (2)	0.80042 (18)	0.0481 (4)	
C10	0.4408 (12)	0.1485 (12)	0.7559 (10)	0.060 (2)	0.349 (12)
H10A	0.4094	0.0537	0.7727	0.072*	0.349 (12)
H10B	0.5466	0.2054	0.8134	0.072*	0.349 (12)
C11	0.417 (2)	0.1330 (19)	0.6029 (7)	0.106 (5)	0.349 (12)
H11A	0.4724	0.0766	0.5728	0.159*	0.349 (12)
H11B	0.4546	0.2283	0.5902	0.159*	0.349 (12)
H11C	0.3106	0.0844	0.5496	0.159*	0.349 (12)
C10'	0.3713 (8)	0.0989 (6)	0.7031 (7)	0.0680 (14)	0.651 (12)
H10C	0.2790	0.0317	0.6284	0.082*	0.651 (12)
H10D	0.4071	0.0459	0.7644	0.082*	0.651 (12)
C11'	0.4937 (6)	0.1734 (7)	0.6475 (8)	0.0788 (16)	0.651 (12)
H11D	0.5166	0.1006	0.5934	0.118*	0.651 (12)
H11E	0.5844	0.2377	0.7232	0.118*	0.651 (12)
H11F	0.4574	0.2288	0.5907	0.118*	0.651 (12)
C12	0.03185 (18)	0.29931 (18)	0.73647 (16)	0.0411 (4)	
C13	−0.1161 (2)	0.1694 (2)	0.6960 (2)	0.0583 (5)	
H13A	−0.1894	0.1721	0.6160	0.087*	
H13B	−0.1549	0.1730	0.7711	0.087*	
H13C	−0.0978	0.0805	0.6753	0.087*	
C14	0.00377 (19)	0.43993 (19)	0.77358 (18)	0.0441 (4)	
H14A	−0.0853	0.4337	0.7010	0.053*	
H14B	−0.0202	0.4466	0.8580	0.053*	
C15	0.2144 (2)	0.66885 (19)	0.91664 (18)	0.0455 (4)	
C16	0.3241 (3)	0.8094 (2)	0.9149 (3)	0.0736 (6)	
H16A	0.3159	0.8900	0.9735	0.110*	
H16B	0.3006	0.8168	0.8221	0.110*	
H16C	0.4263	0.8118	0.9478	0.110*	
C17	0.0227 (2)	0.30512 (19)	0.49921 (17)	0.0458 (4)	
C18	0.1100 (2)	0.2895 (2)	0.4059 (2)	0.0604 (5)	
H18A	0.0421	0.2542	0.3117	0.091*	
H18B	0.1576	0.2214	0.4248	0.091*	
H18C	0.1871	0.3829	0.4205	0.091*	

### Atomic displacement parameters (Å^2^)

**Table:**

	*U*^11^	*U*^22^	*U*^33^	*U*^12^	*U*^13^	*U*^23^
O1	0.0832 (11)	0.0437 (9)	0.1075 (13)	0.0247 (8)	0.0207 (9)	0.0160 (8)
O2	0.0795 (10)	0.0686 (9)	0.0821 (10)	0.0499 (8)	0.0469 (8)	0.0361 (8)
O3	0.0617 (8)	0.0703 (9)	0.0423 (7)	0.0166 (7)	0.0156 (6)	0.0170 (6)
O4	0.0585 (8)	0.0872 (10)	0.0472 (7)	0.0395 (7)	0.0129 (6)	0.0261 (7)
O5	0.0458 (6)	0.0549 (7)	0.0351 (6)	0.0234 (5)	0.0093 (5)	0.0134 (5)
N1	0.0576 (9)	0.0473 (8)	0.0414 (8)	0.0242 (7)	0.0216 (7)	0.0202 (7)
C1	0.0547 (11)	0.0570 (11)	0.0591 (11)	0.0200 (9)	0.0188 (9)	0.0288 (9)
C2	0.0691 (13)	0.0815 (15)	0.0728 (14)	0.0394 (12)	0.0256 (11)	0.0492 (13)
C3	0.0580 (12)	0.0973 (17)	0.0556 (12)	0.0329 (12)	0.0100 (10)	0.0346 (12)
C4	0.0666 (13)	0.0727 (15)	0.0606 (13)	0.0128 (11)	−0.0015 (10)	0.0196 (11)
C5	0.0710 (13)	0.0523 (11)	0.0555 (12)	0.0195 (10)	0.0042 (10)	0.0209 (9)
C6	0.0472 (9)	0.0517 (10)	0.0408 (9)	0.0221 (8)	0.0176 (7)	0.0198 (8)
C7	0.0453 (9)	0.0615 (11)	0.0456 (10)	0.0214 (8)	0.0150 (8)	0.0239 (8)
C8	0.0369 (8)	0.0409 (9)	0.0387 (9)	0.0138 (7)	0.0082 (7)	0.0143 (7)
C9	0.0532 (10)	0.0453 (10)	0.0443 (9)	0.0222 (8)	0.0088 (8)	0.0150 (8)
C10	0.048 (5)	0.059 (5)	0.075 (5)	0.031 (4)	0.016 (4)	0.015 (4)
C11	0.114 (11)	0.117 (10)	0.075 (7)	0.068 (9)	0.013 (6)	−0.013 (6)
C10'	0.077 (4)	0.066 (3)	0.067 (3)	0.044 (3)	0.023 (3)	0.008 (2)
C11'	0.077 (3)	0.097 (4)	0.070 (4)	0.049 (3)	0.030 (3)	0.006 (3)
C12	0.0387 (8)	0.0464 (9)	0.0373 (9)	0.0158 (7)	0.0100 (7)	0.0149 (7)
C13	0.0433 (10)	0.0572 (12)	0.0586 (12)	0.0088 (8)	0.0041 (8)	0.0177 (9)
C14	0.0416 (8)	0.0548 (10)	0.0401 (9)	0.0232 (8)	0.0124 (7)	0.0167 (8)
C15	0.0493 (9)	0.0478 (10)	0.0470 (10)	0.0243 (8)	0.0195 (8)	0.0166 (8)
C16	0.0885 (16)	0.0507 (12)	0.0764 (15)	0.0144 (11)	0.0349 (13)	0.0171 (11)
C17	0.0492 (10)	0.0450 (10)	0.0377 (9)	0.0187 (8)	0.0065 (7)	0.0098 (7)
C18	0.0680 (12)	0.0773 (14)	0.0436 (10)	0.0375 (11)	0.0182 (9)	0.0189 (9)

### Geometric parameters (Å, °)

**Table:**

O1—C9	1.194 (2)	C8—H8	0.9800
O2—C9	1.313 (2)	C10—C11	1.531 (3)
O2—C10	1.423 (8)	C10—H10A	0.9700
O2—C10'	1.493 (5)	C10—H10B	0.9700
O3—C15	1.218 (2)	C11—H11A	0.9600
O4—C17	1.203 (2)	C11—H11B	0.9600
O5—C17	1.335 (2)	C11—H11C	0.9600
O5—C12	1.470 (2)	C10'—C11'	1.521 (3)
N1—C15	1.341 (2)	C10'—H10C	0.9700
N1—C14	1.434 (2)	C10'—H10D	0.9700
N1—H1	0.8600	C11'—H11D	0.9600
C1—C2	1.381 (3)	C11'—H11E	0.9600
C1—C6	1.381 (3)	C11'—H11F	0.9600
C1—H1A	0.9300	C12—C13	1.512 (2)
C2—C3	1.360 (3)	C12—C14	1.521 (2)
C2—H2	0.9300	C13—H13A	0.9600
C3—C4	1.371 (3)	C13—H13B	0.9600
C3—H3	0.9300	C13—H13C	0.9600
C4—C5	1.377 (3)	C14—H14A	0.9700
C4—H4	0.9300	C14—H14B	0.9700
C5—C6	1.371 (3)	C15—C16	1.493 (3)
C5—H5	0.9300	C16—H16A	0.9600
C6—C7	1.506 (2)	C16—H16B	0.9600
C7—C8	1.528 (2)	C16—H16C	0.9600
C7—H7A	0.9700	C17—C18	1.477 (3)
C7—H7B	0.9700	C18—H18A	0.9600
C8—C9	1.508 (2)	C18—H18B	0.9600
C8—C12	1.548 (2)	C18—H18C	0.9600
			
C9—O2—C10	128.9 (5)	H11A—C11—H11C	109.5
C9—O2—C10'	109.6 (2)	H11B—C11—H11C	109.5
C10—O2—C10'	28.2 (3)	O2—C10'—C11'	103.4 (4)
C17—O5—C12	123.96 (13)	O2—C10'—H10C	111.1
C15—N1—C14	123.26 (14)	C11'—C10'—H10C	111.1
C15—N1—H1	118.4	O2—C10'—H10D	111.1
C14—N1—H1	118.4	C11'—C10'—H10D	111.1
C2—C1—C6	120.78 (19)	H10C—C10'—H10D	109.0
C2—C1—H1A	119.6	C10'—C11'—H11D	109.5
C6—C1—H1A	119.6	C10'—C11'—H11E	109.5
C3—C2—C1	120.28 (19)	H11D—C11'—H11E	109.5
C3—C2—H2	119.9	C10'—C11'—H11F	109.5
C1—C2—H2	119.9	H11D—C11'—H11F	109.5
C2—C3—C4	119.68 (19)	H11E—C11'—H11F	109.5
C2—C3—H3	120.2	O5—C12—C13	110.07 (14)
C4—C3—H3	120.2	O5—C12—C14	110.82 (13)
C3—C4—C5	120.0 (2)	C13—C12—C14	109.41 (14)
C3—C4—H4	120.0	O5—C12—C8	101.15 (12)
C5—C4—H4	120.0	C13—C12—C8	113.50 (14)
C6—C5—C4	121.22 (19)	C14—C12—C8	111.67 (14)
C6—C5—H5	119.4	C12—C13—H13A	109.5
C4—C5—H5	119.4	C12—C13—H13B	109.5
C5—C6—C1	118.05 (17)	H13A—C13—H13B	109.5
C5—C6—C7	120.95 (16)	C12—C13—H13C	109.5
C1—C6—C7	121.00 (17)	H13A—C13—H13C	109.5
C6—C7—C8	113.01 (14)	H13B—C13—H13C	109.5
C6—C7—H7A	109.0	N1—C14—C12	115.47 (14)
C8—C7—H7A	109.0	N1—C14—H14A	108.4
C6—C7—H7B	109.0	C12—C14—H14A	108.4
C8—C7—H7B	109.0	N1—C14—H14B	108.4
H7A—C7—H7B	107.8	C12—C14—H14B	108.4
C9—C8—C7	109.42 (14)	H14A—C14—H14B	107.5
C9—C8—C12	109.97 (14)	O3—C15—N1	122.09 (16)
C7—C8—C12	113.48 (13)	O3—C15—C16	122.17 (18)
C9—C8—H8	107.9	N1—C15—C16	115.74 (17)
C7—C8—H8	107.9	C15—C16—H16A	109.5
C12—C8—H8	107.9	C15—C16—H16B	109.5
O1—C9—O2	123.37 (18)	H16A—C16—H16B	109.5
O1—C9—C8	124.07 (18)	C15—C16—H16C	109.5
O2—C9—C8	112.55 (15)	H16A—C16—H16C	109.5
O2—C10—C11	101.9 (8)	H16B—C16—H16C	109.5
O2—C10—H10A	111.4	O4—C17—O5	124.54 (17)
C11—C10—H10A	111.4	O4—C17—C18	124.82 (16)
O2—C10—H10B	111.4	O5—C17—C18	110.64 (15)
C11—C10—H10B	111.4	C17—C18—H18A	109.5
H10A—C10—H10B	109.3	C17—C18—H18B	109.5
C10—C11—H11A	109.5	H18A—C18—H18B	109.5
C10—C11—H11B	109.5	C17—C18—H18C	109.5
H11A—C11—H11B	109.5	H18A—C18—H18C	109.5
C10—C11—H11C	109.5	H18B—C18—H18C	109.5

### Hydrogen-bond geometry (Å, °)

**Table:**

*D*—H···*A*	*D*—H	H···*A*	*D*···*A*	*D*—H···*A*
N1—H1···O4^i^	0.86	2.30	3.074 (2)	149.

Symmetry codes: (i) −*x*, −*y*+1, −*z*+1.
